# Impact of the First and Second Wave of the SARS-CoV-2 Pandemic on Severe Trauma and the Care Structures in the German TraumaNetzwerk DGU^®^

**DOI:** 10.3390/jcm11237036

**Published:** 2022-11-28

**Authors:** Christian Colcuc, Sebastian Fischer, Philipp Leimkühler, Marco Miersbach, Rolf Lefering, TraumaRegister DGU, Dirk Wähnert, Thomas Vordemvenne, Niklas Grüneweller

**Affiliations:** 1 Department of Trauma Surgery and Orthopedics, Protestant Hospital of Bethel Foundation, University Hospital OWL of Bielefeld University, Campus Bielefeld-Bethel, 33617 Bielefeld, Germany; 2 Department of Foot and Ankle Surgery, Berufsgenossenschaftliche Unfallklinik Frankfurt am Main, 60389 Frankfurt, Germany; 3 Institute for Research in Operative Medicine (IFOM), University Witten/Herdecke, 51109 Cologne, Germany; 4 Committee on Emergency Medicine, Intensive Care and Trauma Management (Sektion NIS) of the German Trauma Society (DGU), 10623 Berlin, Germany

**Keywords:** TraumaNetzwerk DGU^®^, COVID-19, level 1, trauma center

## Abstract

(1) Background: The aim of this study was to investigate the effects of the pandemic on transfer rates of severely injured patients within the German TraumaNetzwerk of the DGU. Furthermore, cause of accident, rescue times, and trauma cases are compared to pre-pandemic times. (2) Methods: For this investigation patients documented in the TraumaRegister DGU^®^ from 2018 to 2020 were analyzed. The years 2018 and 2019 served as a comparison to 2020, the first COVID-19 pandemic year. All primary admissions and transfers were included if treated on an intensive care unit. (3) Results: Demographics (age, sex) and injury severity in 2020 were comparable with 2018/2019. In 2020, a significant decrease (3.7%) in car accidents was found. In contrast, a significant increase (3.2%) in bicycle accidents was seen. During the second wave, there was a significant burden of COVID-19 patients on hospitals. In this time, we found a significant increase in early transfers of trauma patients primarily from small level 3 to large level 1 centers. There was also a small but significant increase in rescue time, especially during the 2nd wave. (4) Conclusions: Our data confirm the importance of the network structures established in the TraumaNetzwerk DGU^®^, especially during the pandemic. The established structures allow smaller hospitals to spread their resources and prevent internal collapse. Therefore, the structures of the TraumaNetzwerk DGU^®^ play a prominent role in stabilizing the healthcare system by helping to maintain both surgical and critical care capacity and providing adequate emergency care.

## 1. Introduction

To date, more than six million people have died from COVID-19 worldwide. This makes the COVID-19 pandemic the third deadliest mass viral disease in recent history, with only the Spanish flu and AIDS having claimed more lives [1].

The record infection figures of the ‘4th wave’ (virus variant) and the resulting tense situation in the intensive care units in some federal states in Germany made it necessary to transfer intensive care patients to other federal states on a larger scale with the participation of the Air Force [2].

However, in addition to limited critical care capacity, trauma patient care is also a major challenge. In addition, there are geographic and infrastructural differences in terms of care options. Reasons for this are differences in the staffing and equipment of hospitals. To improve trauma care nationwide the German Trauma Society (DGU) had initiated the TraumaNetzwerk DGU^®^ (TNW) project. Commonly defined standards of care, the integration of regional cooperations, and the division of hospitals into local, regional, and supraregional trauma centers make it possible for the TNW to structure and influence the care of severely injured patients within a nationwide trauma system [3].

While the overall number of traumas decreased, there appeared to be a greater overall concentration in level 1 trauma centers during the pandemic [4].

Another study from a level 1 trauma center was able to show that there was a 50% increase in polytrauma patients. In addition, the number of bed days of trauma surgery patients in the ICU and IMC increased by 90% at the start of the pandemic in March 2020 compared to the same period the previous year [5].

This study investigated the role of the network structure within the TraumaNetzwerk DGU^®^ during the 1st and 2nd wave and how the transfer structures between the trauma centers changed during the 1st and 2nd wave. In addition, the number of trauma cases, rescue time, severity of injury, and accident mechanism were examined and compared to previous years.

## 2. Materials and Methods

The TraumaRegister DGU^®^ of the German Trauma Society (Deutsche Gesellschaft für Unfallchirurgie, DGU) was founded in 1993. The aim of this multicenter database is a pseudonymized and standardized documentation of severely injured patients.

Data are collected prospectively in four consecutive time phases from the site of the accident until discharge from hospital: (A) pre-hospital phase, (B) emergency room and initial surgery, (C) intensive care unit, and (D) discharge. The documentation includes detailed information on demographics, injury pattern, comorbidities, pre- and in-hospital management, course on intensive care unit, relevant laboratory findings including data on transfusion, and outcome of each individual. The inclusion criterion is admission to hospital via emergency room with subsequent ICU/ICM care or reaching the hospital with vital signs and death before admission to ICU.

The infrastructure for documentation, data management, and data analysis is provided by AUC—Academy for Trauma Surgery (AUC—Akademie der Unfallchirurgie GmbH), a company affiliated with the German Trauma Society. The scientific leadership is provided by the Committee on Emergency Medicine, Intensive Care and Trauma Management (Sektion NIS) of the German Trauma Society. The participating hospitals submit their data pseudonymized into a central database via a web-based application. Scientific data analysis is approved according to a peer review procedure laid out in the publication guideline of the TraumaRegister DGU^®^.

The participating hospitals are primarily located in Germany (90%), but a rising number of hospitals in other countries contribute data as well (at the moment Austria, Belgium, China, Finland, Luxembourg, Slovenia, Switzerland, the Netherlands, and the United Arab Emirates). Currently, approximately 30,000 cases from 650 hospitals are entered into the database per year.

Participation in the TraumaRegister DGU^®^ (TR-DGU) is voluntary. For hospitals associated with the TraumaNetzwerk DGU^®^, however, the entry of at least a basic dataset is obligatory for reasons of quality assurance.

The present study (TR-DGU Project ID: 2020-056) and manuscript were approved according to the publication guidelines of the TR-DGU.

### 2.1. Patients

Severely injured patients treated in a German hospital and documented in TR-DGU from 2018 to 2020 were analyzed for this study. The years 2018 and 2019 served as a comparison to 2020, the first COVID-19 pandemic year. Due to the fact that restrictions were imposed on the population in the context of the pandemic for the first time in Germany in March 2020, the evaluation of the data was carried out for all three years from the 10th calendar week. The restrictions included, among others, contact restrictions, wearing masks, keeping distance from other people, and the closure of restaurants. Further analysis of the COVID-19 year 2020 was based on the pandemic. For this purpose, the following periods were defined: 1st wave (10th to 20th calendar week), summer plateau (21st to 39th calendar week) and 2nd wave (40th to 52nd calendar week).

The basic collective of the TraumaRegister DGU^®^ used for the annual audit reports was considered here. This patient group is defined as all severe trauma cases with maximum Abbreviated Injury Scale (mAIS) severity of 3 or more, or cases with mAIS 2 treated on an intensive care unit. All cases from Germany were included; no further exclusion criteria have been used.

### 2.2. Data Analysis and Statistics

Descriptive data analysis was performed using SPSS (version 27, IBM, Armonk, NY, USA). Mean and standard deviation was used for metric data, and percentage for counts. Due to the large number of patients per year, a formal statistical testing of observed differences was avoided. This would have resulted in highly significant results even in non-relevant differences. The detectable difference in groups of 20,000 cases each is <1%.

For changes in number of patients, 95% confidence intervals (CI) were calculated based on the Poisson distribution. The pre-hospital time from accident to hospital admission has also been analyzed with a multiple linear regression analysis, and adjusted effects for time of year (phases) and year were presented with 95% CI.

## 3. Results

Table 1 shows the comparison of the 2020 COVID-19 pandemic population with those of 2018 and 2019.

Comparing the demographics of trauma patients in 2020 to those of trauma patients in the previous two years shows an increase in average age by 1 year. Fittingly, the proportion of patients with a pre-injury ASA score of 3–4 also increases by 3.5%. The sex distribution (30% female/70% male) is constant over the years. The mean injury severity, as measured by the ISS, also remains constant over the observation period.

In 2020, there is an increase in higher degree injuries (AIS 3+) to the head and thorax. The analysis of the causes of accidents shows a significant decrease of about 3.7% in car accidents during the pandemic year 2020 compared to 2019 and 2018. In contrast, there is a relevant increase of about 3.2% in bicycle accidents. When all traffic accidents are considered together, there is a slight decrease of about 1.4% in 2020. On the other hand, there is a slight increase in suicides (+0.6%, Table 1).

When analyzing the trauma patient flows during the different phases of the pandemic in 2020, it becomes apparent that there were obvious changes during the 2nd wave. During this wave, the maximum incidence in Germany was 210 and there was a significant burden of COVID-19 patients on hospitals. During this time, there was a relevant increase in early transfer (<48 h after admission) of trauma patients from primarily level 3 (approximately +5%) trauma centers to primarily level 1 centers (Table 2).

Evaluating the number of trauma cases during the pandemic waves in 2020, it is clear that the interdiction measures led to a significant decrease in the number of cases.

During the summer months (calendar weeks 21–39), the incidence in Germany dropped to 16 but trauma numbers increased significantly by 4.8% at this time (Table 3).

Another effect of the load on clinics with COVID-19 patients can be seen in rescue time (time from accident event to arrival at the clinic). Compared to the spring (1st wave), there was a slight decrease of −0.2 min (95% CI −0.9–0.5) during the summer plateau and an increase of 1.3 min during winter (95% CI 0.5–2.1) (Table 4).

## 4. Discussion

The COVID-19 pandemic is affecting all medical specialties and all sectors of medical care, from pre-hospital rescue to rehabilitation. The main finding of this paper is that the COVID-19 pandemic had an impact on trauma patient transfer within network structures and thus on the care of severely injured patients in Germany.

This was especially the case during the 2nd wave in 2020 when a large burden of COVID-19 patients admitted to hospitals, as well as a slightly increased transfer of trauma patients to level 2 and level 1 centers was shown.

During this time, there was an increase in early transfer of trauma patients from primarily level 3 (approximately +5%) trauma centers to primarily level 2 (+1.5%) and level 1 centers (+1.5%). Additionally, we found delayed rescue times in this period. We were able to show that the contact restriction measures also had an impact on the number of accident injuries and the causes of accidents. Thus, there was a decrease in the total number of serious injuries during the lockdown periods. When analyzing accident type there was a 3.2% increase in bicycle accidents in 2020 compared to reference the period 2018/19 and a 3.7% decrease in car accidents.

In their study and the evaluation of the eTrauma management platform (Open Medical, London, UK), Sephton et al.’s results also showed a decrease in car accidents (−7%), as well as a significant decrease in sports injuries. Equivalent to the increase in bicycle accidents shown in our study, the authors report an increase in accidents with pushbikes and scooters in northwest London [6].

Both the nationwide lockdown and the increase in those who switched to working from home as part of the lockdown resulting in less traffic could adequately explain the decrease in traffic accidents. The additional restriction of use and closure of sports facilities and sporting events account for the decrease in sports injuries found by Saphton et al. and, likewise, for the increase in bicycle accidents we found.

Due to the fact that sporting activity could only take place in private, the sales figures of the German bicycle industry increased by more than 10% in the first half of 2020 compared to the same period of the previous year [7].

The increase in head and thorax injuries found in our data can be explained by the increased activity and mobility with bicycles in 2020.

The reciprocal development between COVID-19 incidences and trauma figures in Germany also find their origin in the overall reduced activity of each individual within the restricted period.

There was an increase in the total rescue time evaluated across Germany (time from accident event to arrival at the hospital), particularly in the 2nd wave of the pandemic. The total rescue time increased by about 2 min in contrast to the previous times.

This observation is supported by the working group of Driessen et al. from a multicenter observational cohort study based on the Dutch Nationwide Trauma Registry. They showed that the total pre-hospital time was significantly longer for all periods in 2020: 54 min (IQR 44–65) during 1st wave, 53 min (IQR 43–64) during interbellum period, and 54 min (IQR 45–66) during 2nd wave [8]. Different reasons suggest effects of the pandemic on pre-hospital rescue time.

The increased pre-hospital rescue time could be considered a consequence of increased infection control during the 1st and 2nd waves. A retrospective cohort study compared trauma patients transported via EMS to six US level 1 trauma centers admitted 1 January 2019–31 December 2019 and 16 March 2020–30 June 2020 found no difference in total pre-hospital time during the SARS-CoV-2 pandemic waves.

They showed that transportation time was significantly shorter during COVID-19 compared to 2019. This may have been partially due to social distancing guidelines reducing the number of people on the road [9].

Due to the varying quality of care for severely injured patients in Germany, the German Trauma Society launched the TraumaNetzwerk DGU^®^ initiative in 2006 [10,11]. The reasons for these differences in quality are geographical and infrastructural differences between the federal states and regions in Germany, as well as differing treatment concepts and internal equipment of the individual hospitals involved in the care of severely injured patients.

For example, analysis from the United States showed that the introduction of regionalized trauma systems reduced the rate of preventable deaths in severely injured patients by 50% [12,13].

The designations level 1 and level 2 trauma center, as well as level 3 trauma center, were developed based on the equipment of the hospitals from the aspect of severe injury care,. The implementation of trauma networks, the release of the S3 polytrauma guidelines, and the DGU “Weißbuch” have contributed to more structured management of most severely injured patients [14].

Based on these guidelines, transfer strategies between trauma centers are clearly regulated. In the present study, these transfer structures were analyzed during the 1st and 2nd waves of the pandemic. Among other things, it was investigated whether the graduated structure of the trauma networks led to an increased use of higher levels of trauma care during the pandemic.

Most of the published studies showed no differences or a decrease in trauma cases during the first lockdown period [15,16].

In Germany, Wähnert et al. described the effects of the first pandemic wave on a German level 1 trauma center [5]. They showed a significant increase in the number of polytrauma cases in the investigated German trauma center. This confirms the data presented here, which show a slight increase in early transfers from level 3 to level 2 and level 1 centers within the German trauma network, particularly during the 2nd wave. These data confirm the importance of trauma networks, especially during the pandemic. The established structures allow smaller hospitals to spread their resources and prevent internal collapse. Trauma networks therefore play a prominent role in stabilizing the health care system by maintaining both surgical and critical care capabilities and providing adequate emergency care.

This study is limited by the national specificity of the German trauma network structures so the data cannot be generalized.

Furthermore, a limitation of our study is the rather short comparison period (2 years); this is due to a change in the data collection of the TraumaRegister DGU^®^.

Due to the large number of cases evaluated, the statistical evaluation is rather descriptive, as even small differences can reach significance. The evaluation covers the whole of Germany, so regional differences and special features (Wähnert et al., 2021 [5]) are not taken into account in this study.

The different impacts of the respective waves on the health system varies and are not recorded.

## 5. Conclusions

The 2 min increase in rescue time for accidents in our data can currently only be explained by increased infection control efforts. A separate study should focus on how to not have potentially life-critical increased rescue times even during a pandemic. Our data confirm the importance of the network structures established in the TraumaNetzwerk DGU^®^, especially during a pandemic. Level 3 trauma centers, in particular, should take advantage of these network structures by early transfer to preserve their capacities and avoid internal collapse. The structures of the TraumaNetzwerk DGU^®^, therefore, play an outstanding role in stabilizing the healthcare system.

## Figures and Tables

**Table 1:** Demographic data of the study population (ISS Injury Severity Score, ASA American Society of Anesthesiologists, AIS Abbreviated Injury Scale).

	2018 (*n* = 23,582)		2019 (*n* = 20,967)		2020 (*n* = 20,846)	
sex (male)	70.4%		69.1%		70%	
	mean	standard deviation	mean	standard deviation	mean	standard deviation
age	53.0	22.5	54.0	22.4	54.3	22.4
ISS	17.6	11.4	17.5	11.4	17.8	11.4
ASA 3–4	18.5%		19.2%		22.0%	
relevant head injury (AIS 3+)	32.6%		32.6%		33.7%	
relevant thorax injury (AIS 3+)	37.4%		37.3%		38.7%	
relevant abdominal injury (AIS 3+)	9.3%		9.2%		8.9%	
relevant extremities injury (AIS 3+)	22.7%		23.4%		23.1%	
trauma mechanism						
car	19.6%		19.3%		15.8%	
motorcycle	14.5%		13.7%		13.9%	
bike	11.0%		11.5%		14.4%	
pedestrian	4.6%		4.8%		3.7%	
all traffic accidents	50.9%		50.8%		49.4%	
other traffic accidents	1.7%		1.9%		1.8%	
high fall	15.1%		14.3%		15.4%	
low fall	25.6%		26.6%		26.9%	
blunt hit	2.9%		3.0%		2.2%	
gunshot	0.4%		0.4%		0.4%	
stabbing	2.0%		1.8%		2.1%	
others	2.6%		2.7%		3.3%	
suicide	3.9%		3.9%		4.5%	
violence	2.4%		2.0%		2.4%	

**Table 2:** Trauma patient flow during the different phases of the pandemic.

		1st Pandemic Wave	Summer Plateau	2nd Pandemic Wave
Level 1				
	primary	88.9%	89.2%	87.2%
	transfer in	9.4%	9.4%	11.0%
	early transfer out (<48 h after admission)	1.7%	1.4%	1.8%
Level 2				
	primary	87.7%	88.3%	86.5%
	transfer in	3.3%	1.8%	3.1%
	early transfer out (<48 h after admission)	8.9%	9.8%	10.4%
Level 3				
	primary	83.0%	83.6%	78.4%
	transfer in	1.1%	0.9%	0.7%
	early transfer out (<48 h after admission)	15.9%	15.5%	20.9%

**Table 3:** Trauma cases during the different phases of the pandemic in 2020 compared to 2019 for all primary patient admissions.

	Max. Incidence SARS-CoV-2	2019	2020	Relative Change	95% Confidence Interval
1st pandemic wave (week 10–20)	43	4858	4708	−3.1	−0.3; −5.8
summer plateau (week 21–39)	16	9821	10,250	+4.4	2.4; 6.4
2nd pandemic wave (week 40–52)	210	4811	4578	−4.8	−2.0; −7.6

**Table 4:** Time (minutes (standard deviation)) from accident to arrival at the hospital for all primary patient admissions. Mean change compared to 2018 with 95% confidence intervals.

	1st Pandemic Wave	Summer Plateau	2nd Pandemic Wave	Mean Change (min) 95% Confidence Interval
2018	64.6 (31.8)	64.3 (30.8)	65.5 (32.1)	baseline
2019	65.3 (31.9)	65.1 (30.9)	66.8 (32.4)	+0.9 0.2–1.6
2020	66.7 (31.4)	66.6 (32.3)	68.1 (34.4)	+2.4 1.7–3.1

## Data Availability

The data remain in the possession of the TraumaRegister DGU^®^. Each participant of the registry can request data there.
